# Supplementary material for: Atopobium vaginae and Prevotella bivia Are Able to Incorporate and Influence Gene Expression in a Pre-Formed Gardnerella vaginalis Biofilm
Source: Pathogens. 2021 Feb 20;10(2):247. doi: 10.3390/pathogens10020247 (PMC7924186; doi:10.3390/pathogens10020247)
Supplement: Supplementary file 1 [file pathogens-10-00247-s001.zip › supplementarty proof/Supplementary Figure 3 v1.docx]

**A**


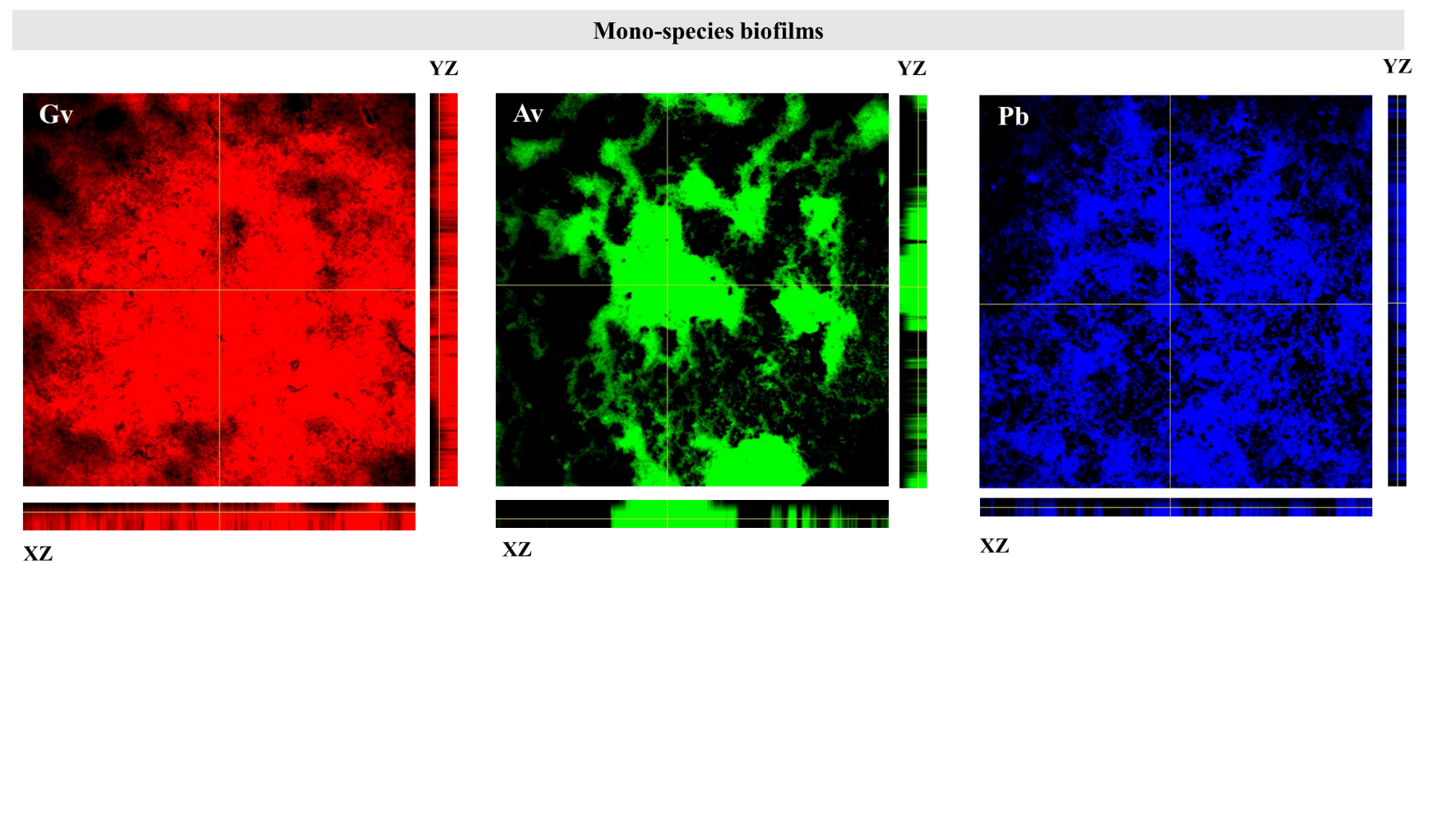


**B**


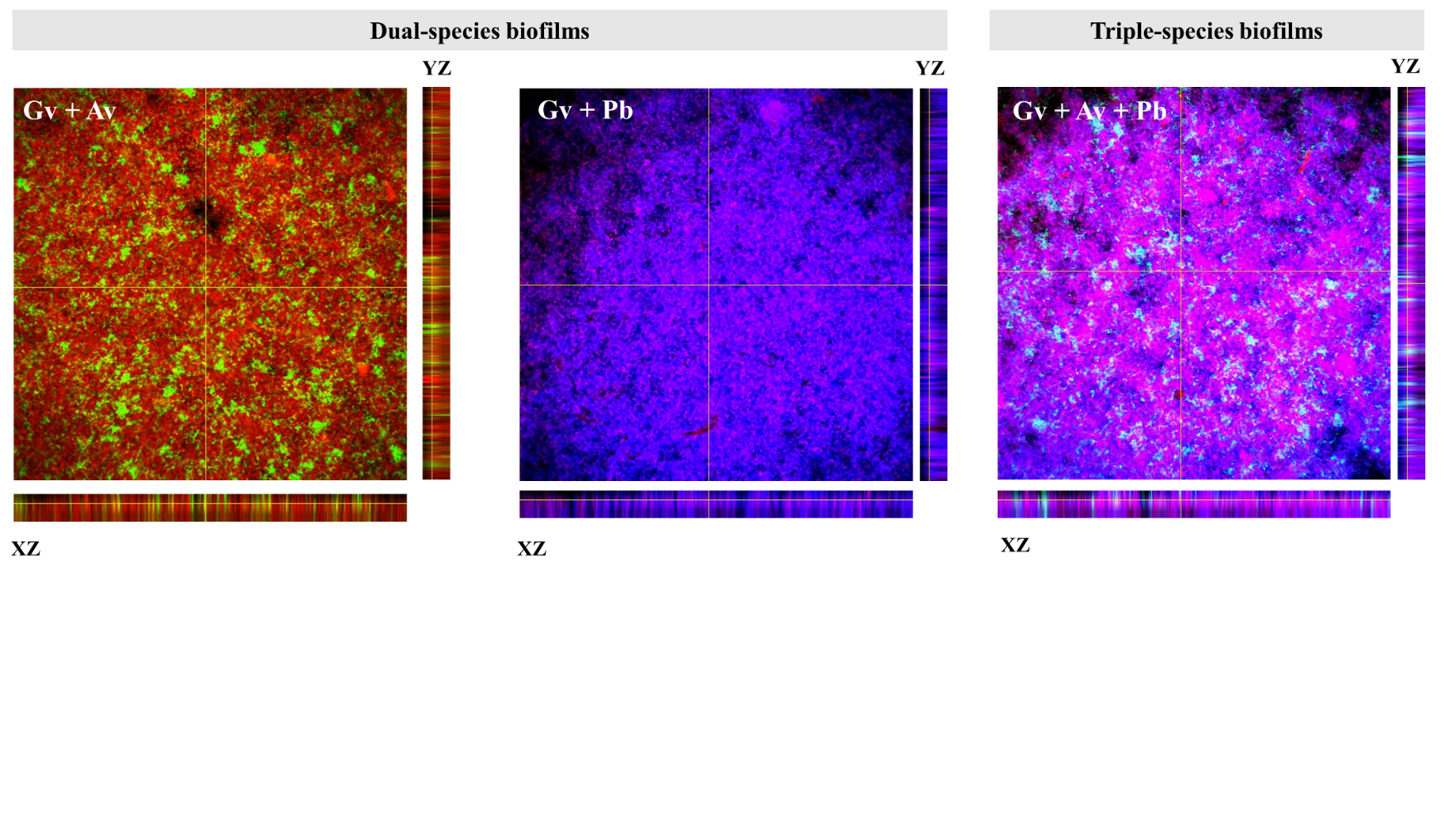


**Supplementary Figure 3**. An example of orthogonal views of BV-associated biofilms by confocal laser scanning microscopy (CLSM). **(A)** Mono-species biofilms of Gv, Av and Pb. **(B)** Dual- and triple-species biofilms. Gv and Av cells were differentiated by hybridization with PNA Gard162 (red/purple color when coupled with DAPI) and AtoITM1 probes (green/blue-green color when coupled with DAPI), respectively, while Pb was differentiated by DAPI (blue color). Abbreviations: *A. vaginae* (Av), *G. vaginalis* (Gv), and *P. bivia* (Pb).
