# Supplementary material for: Atopobium vaginae and Prevotella bivia Are Able to Incorporate and Influence Gene Expression in a Pre-Formed Gardnerella vaginalis Biofilm
Source: Pathogens. 2021 Feb 20;10(2):247. doi: 10.3390/pathogens10020247 (PMC7924186; doi:10.3390/pathogens10020247)
Supplement: Supplementary file 1 [file pathogens-10-00247-s001.zip › supplementarty proof/SupplementaryFigure1.docx]

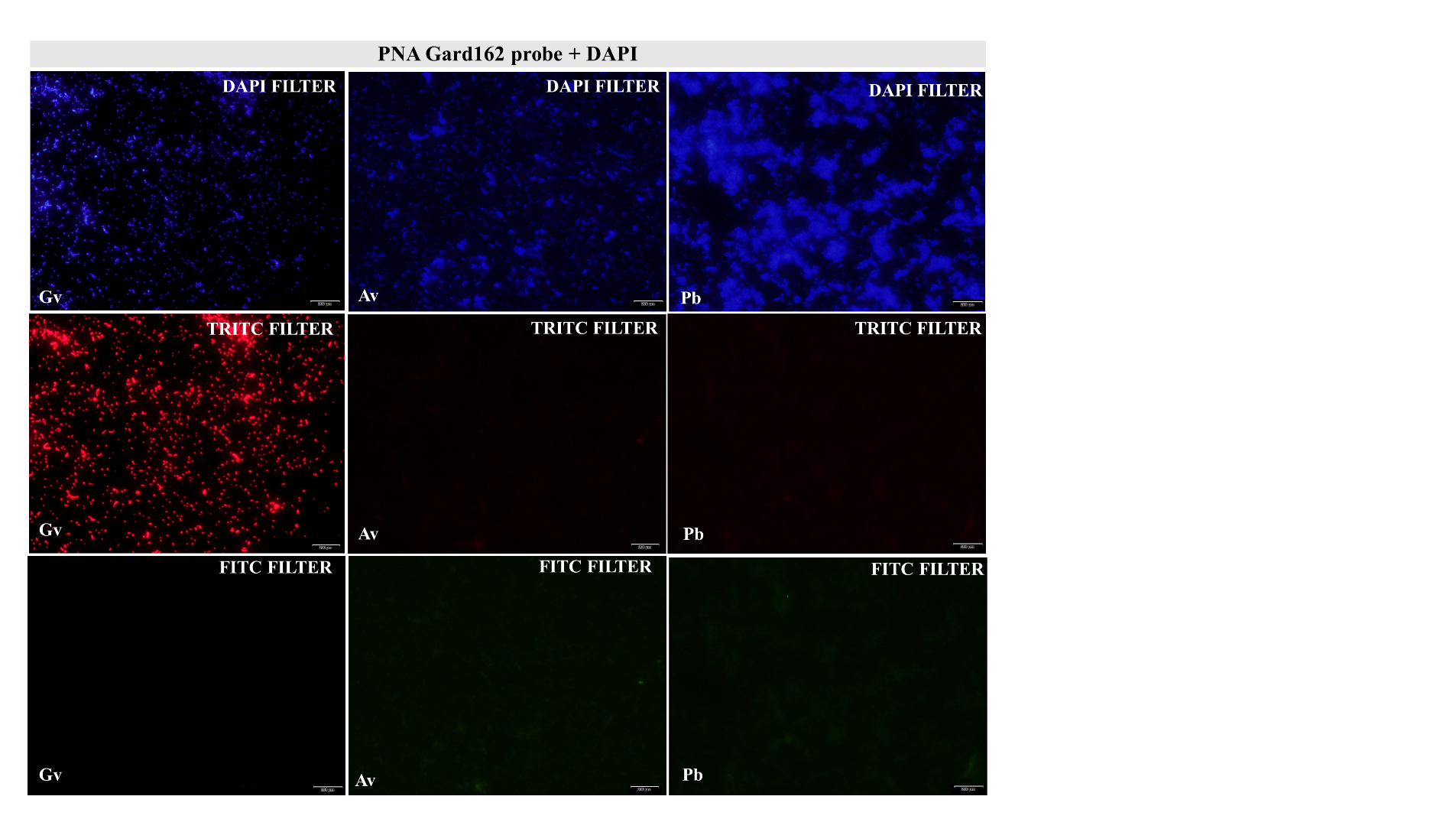


**Supplementary Figure 1**. An example of fluorescence microscopy pictures representing the specificity of Gard162 probe. Gv cells were differentiated by hybridization with PNA Gard162 probe (red color). DAPI served as a control that labeled all the bacterial species. Abbreviations: *A. vaginae* (Av), *G. vaginalis* (Gv), and *P. bivia* (Pb).
