# Supplementary material for: Atopobium vaginae and Prevotella bivia Are Able to Incorporate and Influence Gene Expression in a Pre-Formed Gardnerella vaginalis Biofilm
Source: Pathogens. 2021 Feb 20;10(2):247. doi: 10.3390/pathogens10020247 (PMC7924186; doi:10.3390/pathogens10020247)
Supplement: Supplementary file 1 [file pathogens-10-00247-s001.zip › supplementarty proof/SupplementaryTable1.docx]

**Table S1** Primers used in qPCR experiments

| Target gene | Gene description | Primer sequence (5’ to 3’) | T _melting_ (ºC) | Efficiency ^a^ (%) | Amplicon size (bp) |
| --- | --- | --- | --- | --- | --- |
| *16S RNA* | 16S ribosomal RNA of *G. vaginalis* | Fw TGAGTAATGCGTGACCAACC  Rv AGCCTAGGTGGGCCATTACC | 55.2  59.3 | 100 | 167 |
| *vly* | Thiol-activated cytolysin vaginolysin | Fw GAACAGCTGGGCTAGAGGTG  Rv AATTCCATCGCATTCTCCAG | 60.01  60.04 | 100 | 153 |
| *sld* | Sialidase | Fw CCGAATTTGCGATTTCTTCT  Rv CGTACGGAAGTTTTGGAAGC | 54.00  58.00 | 86 | 189 |
| *HMPREF0424_0821* | Glycosyltransferase, group 2 family protein | Fw CAACGAAGGCATAGGTTTCC  Rv GCGCTTGGAACTGCTTTAAC | 59.57  60.02 | 100 | 156 |
| *HMPREF0424_1122* | Multidrug resistance ABC transporter | Fw CAGCACCTGTAGCTCCAACA  Rv TGGCTCAAGAGATTGTGTGC | 60.05  59.99 | 89 | 195 |
| *HMPREF0424_0156* | Bacitracin transport ATP-binding protein BcrA | Fw CCGACCGCATACCTATTTTG  Rv GCAAGACGGTCTCCAAACTC | 60.34  59.85 | 90 | 178 |
| *HMPREF0424_1196* | LPXTG-motif cell wall anchor domain-containing protein | Fw TGCAAAGACAGGCGATAGTG  Rv TAATCGTTGCGGTTGTTTCA | 60.00  60.11 | 99 | 173 |

^a^ PCR amplification efficiency (E) for each gene was determined from the slope of a standard curve (E =10 [-1/slope]), generated with a 10-fold dilution series of cDNA.
